# Brain Computed Tomography Angiography as an Ancillary Test in the Confirmation of Brain Death

**DOI:** 10.7759/cureus.1491

**Published:** 2017-07-19

**Authors:** Homa Sadeghian, Mohammad Ali Raeisi, Parviz Dolati, Rouzbeh Motiei-Langroudi

**Affiliations:** 1 Radiology, Massachusetts General Hospital/Harvard Medical School; 2 Neurology, Pastor Hospital, Bam University of Medical Sciences; 3 Neurosurgery, Beth Israel Deaconess Medical Center, Harvard Medical School

**Keywords:** brain death, ancillary test, computed tomography angiography, diagnosis

## Abstract

Introduction

Brain death (BD) is the irreversible termination of the functioning of the brain. The diagnosis should be first made by clinical criteria and confirmed by using paraclinical confirmatory techniques (ancillary tests). While conventional brain angiography remains the standard method of choice, computed tomography angiography (CTA) has emerged as an alternative method. In this study, we tried to evaluate the accuracy of CTA for the diagnosis of BD.

Methods

In this study, we included nine patients with a clinical diagnosis of BD, confirmed by electroencephalography (EEG). CTA was then performed to compare the results.

Results

The most frequent cause for BD was multiple trauma (7/9) in our patients, followed by aneurysm rupture and brain infarct. CTA examination in all patients showed opacification of extracranial arteries and major branches of external carotid artery (ECA), including superficial temporal arteries (STAs), while no opacification was observed in the internal carotid arteries (ICA) including and beyond the cavernous segment, middle cerebral arteries (MCAs), anterior cerebral arteries (ACAs), distal vertebral arteries (VAs), and basilar artery (BA). Moreover, no opacification was observed in the internal cerebral veins (ICVs) or great cerebral vein (GCV).

Conclusion

The accuracy rate of CTA in the detection of intracranial circulatory arrest was 100%. CTA examinations confirmed BD diagnoses in all patients who had clinical and EEG BD diagnoses, and no confliction between CTA findings and clinical diagnoses was observed.

## Introduction

Brain death (BD) is a state of irreversible termination of the functioning of the entire brain. The diagnosis of BD should be first made on the basis of clinical criteria [1-2]. This diagnosis should be accurately made as the BD population can potentially serve in organ donation [1].

The protocol to confirm BD in many countries not only requires a clinical diagnosis but also the use of paraclinical confirmatory techniques (ancillary tests), which include electroencephalography (EEG), evoked potentials, transcranial Doppler (TCD), and conventional brain angiography [3]. The objective of the ancillary tests is to demonstrate the absence of cerebral electrical activity or cerebral circulatory arrest [1].

While conventional brain angiography with a catheter remains the standard method of choice, computed tomography angiography (CTA) has emerged as an alternative method [1, 3-4]. CTA is easily accessible in almost all hospitals, has a high spatiotemporal resolution, is operator independent, and inexpensive [2, 5-6]. However, some studies have shown less promising results for CTA in the confirmatory diagnosis of BD, as it was associated with a considerable false-positive and false-negative burden and did not recommend it as a means of BD diagnosis [7-8]. To solve the discrepancy between the results of CTA and those seen in standard ancillary tests, some studies suggest to confine the CTA imaging feature to specific arterial and venous branches [9] or add CT perfusion findings for a diagnosis of BD [10-11].

In the current study, we first confirmed BD in patients by clinical criteria and EEG. We then performed brain CTA in these patients and compared its yield of diagnosis and accuracy to the EEG, trying to advocate a highly accurate and specific CTA finding. The goal of our study was to evaluate the accuracy of cerebral CTA for the diagnosis of BD.

## Materials and methods

Between February 2015 and August 2015, nine patients were enrolled in the study. First, BD was suggested by a nurse with special training for the diagnosis of BD. Repeating the examination after six to 12 hours, the clinical diagnosis was confirmed by both an anesthesiologist and a neurologist, blind to the results of each other’s evaluation. The inclusion criteria were: 1) unresponsive coma with a Glasgow Coma Scale (GCS) of 2/10 (all patients were intubated and under mechanical ventilation); 2) absence of brainstem reflexes (mydriatic pupils with no reaction to light, no corneal, cough, and oculocephalic reflexes, no reaction to trigeminal pain stimuli); 3) absence of central spontaneous respiratory drive assessed by apnea test. The exclusion criteria were: 1) abnormal serum electrolytes or acid-base state; 2) prescription of any kind of central nervous system (CNS) or suppressant drugs (thiopental, fentanyl, midazolam, other benzodiazepines). Patients who were diagnosed to have BD by both physicians would then undergo a standard EEG based on accepted guidelines for the diagnosis of BD [12-13]. After confirmation of BD by both clinical examination and EEG, CTA was performed following standard protocols [3, 14] using a 16-slice CT (Siemens, Germany). The CTA images were independently evaluated by a radiologist and a neurosurgeon. The results of CTA were then compared with EEG results.

The patients’ families gave their written consent to participate in the study. All procedures were performed in accordance with local ethical committee of Medical University.

## Results

In the study period, nine patients fulfilled both clinical (GCS 2/10 in intubated patients under mechanical ventilation, no brainstem reflex, positive apnea test) and EEG criteria (isoelectric line for the entire 30 minutes recording) for the diagnosis of BD (Table 1).

**Table 1:** Patients Confirmed to Have Brain Death by Clinical and Electroencephalography Criteria BD: brain death; CT: computed tomography; CVA: cerebrovascular accident; EDH: epidural hematoma; fx: fracture; ICH: intracerebral hematoma; ICU: intensive care unit; IVH: intraventricular hemorrhage; SAH: subarachnoid hemorrhage; SDH: subdural hematoma

*Age*	*Sex*	*Initial Brain CT findings*	*Duration of ICU stay prior to BD (days)*	*BD cause/mechanism*
32	M	EDH, skull base fx, sulcal and cisternal effacement	3	Multiple trauma
18	M	ICH, SDH, sulcal and cisternal effacement	1	Multiple trauma
35	F	SAH, IVH, edema	2	Aneurysmal SAH
42	F	SDH, skull fx, sulcal and cisternal effacement	2	Multiple trauma
22	M	sulcal and cisternal effacement	1	Multiple trauma
57	M	ICA infarct	3	Ischemic CVA
25	F	SDH, sulcal, and cisternal effacement	1	Multiple trauma
35	M	Contusions, sulcal and cisternal effacement	2	Multiple trauma
45	M	SDH, skull fx, sulcal and cisternal effacement	1	Multiple trauma

The mean patient age was 34.6 years. The most frequent cause for BD was multiple trauma (seven out of nine) in our patients, followed by aneurysm rupture and brain infarct.

**Figure 1:**
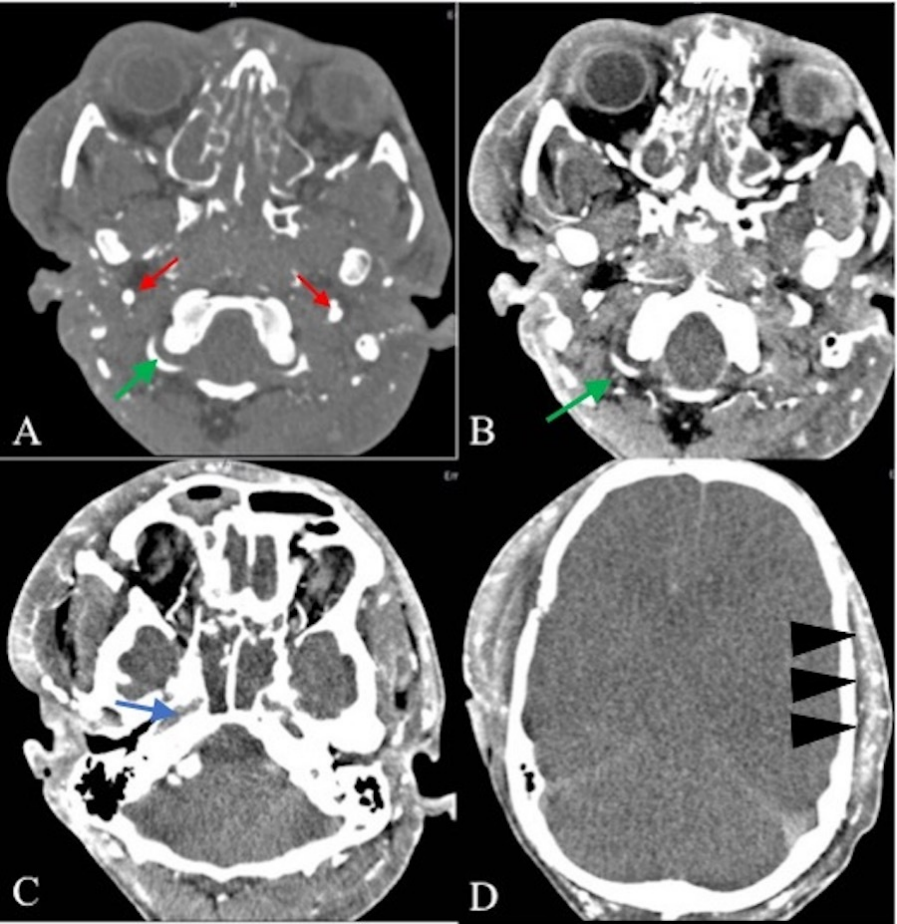
Axial CT angiography of intracranial and extracranial arteries in different levels Consecutive images show flow in the cervical segments of the internal carotid (A, red arrow) and vertebral arteries (A, B, green arrow), which do not continue to the petrous carotid (C, blue arrow) and intradural vertebral arteries (C), and thereafter (D). The extracranial arteries are still opacified (D, arrowheads).

CTA examination in all patients showed opacification of extracranial arteries and major branches of the external carotid artery (ECA), including superficial temporal arteries (STAs), showing the proper administration of contrast media and reliability of CTA studies. In three patients, no opacification was observed in internal carotid arteries (ICA) after the cervical segment, middle cerebral arteries (MCAs), anterior cerebral arteries (ACAs), basilar artery (BA), or vertebral arteries (VA) in their entire intradural segments (‘complete intracranial non-filling’) (Figures 1).

**Figure 2:**
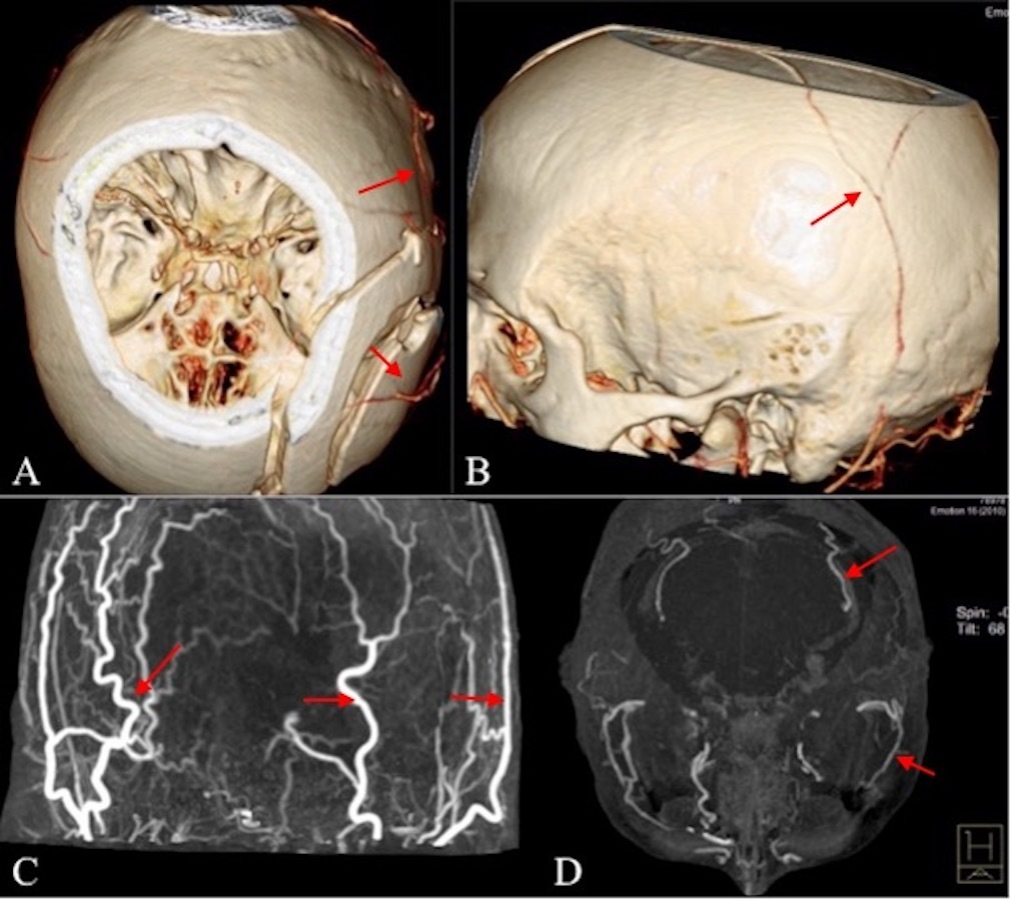
CT angiography with 3D reconstruction Images in four different patients show preservation of external cranial flow (A, B, C, D, red arrows) with no internal cranial opacification (A, C, D).

Although the complete intracranial non-filling was seen, extracranial flow was still observed in some cases (Figure 2).

**Figure 3:**
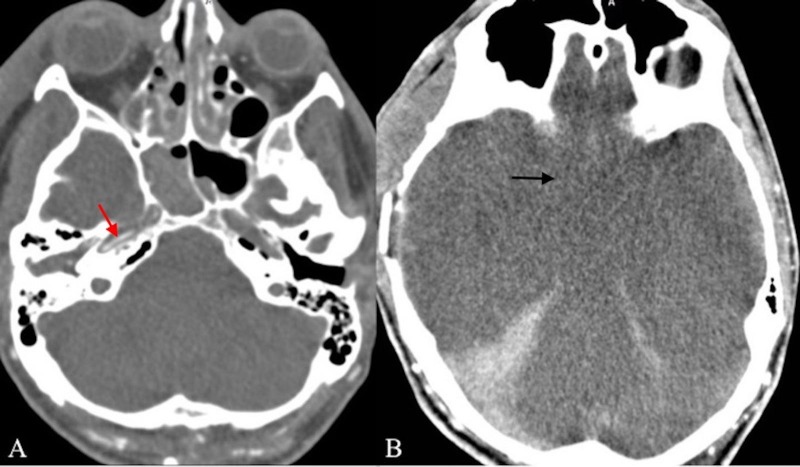
Axial CT angiography of intracranial arteries in two different levels. Images show flow in the petrous segment of internal carotid arteries (A, red arrow), which do not continue thereafter (B, black arrow). There is no flow in the posterior circulation.

In six patients, there was some remnant unilateral ICA flow until the petrous segments, which abruptly terminated thereafter. There was no opacification in MCAs, ACAs, VAs, and BA, however (Figure 3).

**Figure 4:**
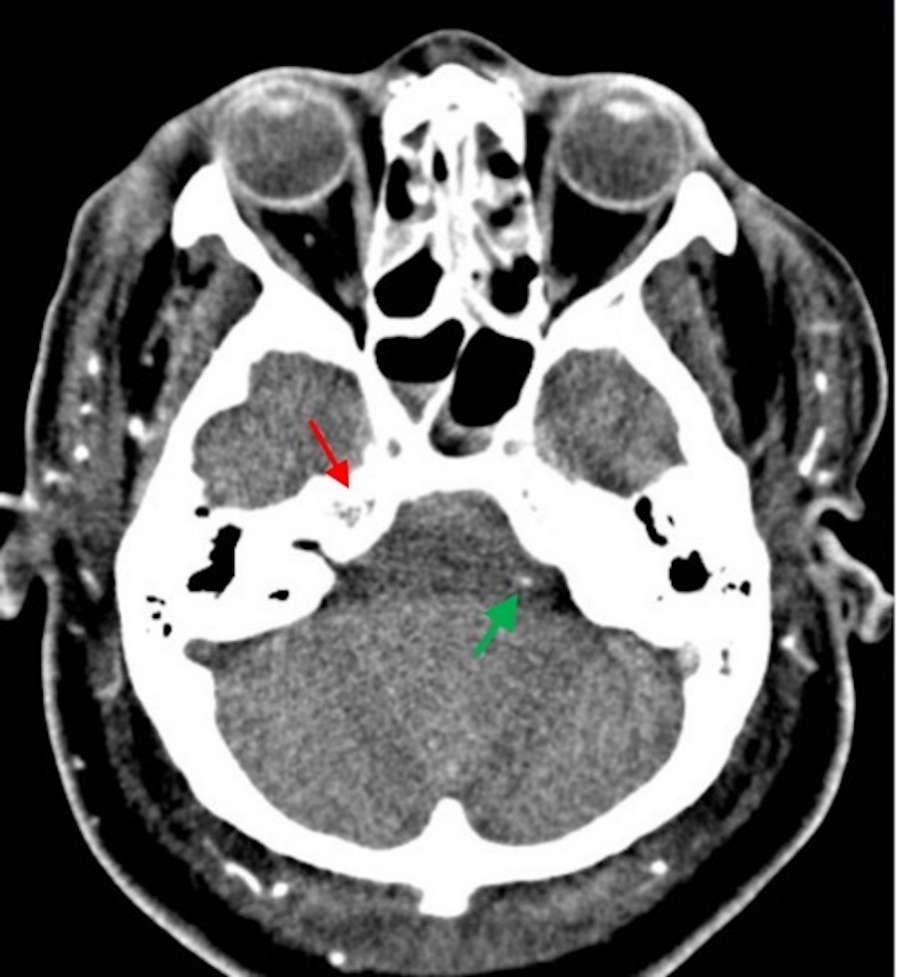
Axial CT angiography of intracranial arteries. Image shows the flow in the petrous segment of internal carotid (red arrow) and proximal intradural vertebral arteries (green arrow) but not thereafter.

Among these six patients, two had also minor unilateral filling in a short length of intradural VA (proximal V4) (Figure 4).

Again, no opacification was observed in MCAs, ACAs, and BA. In all nine patients, no opacification was observed in the internal cerebral veins (ICVs) or great cerebral vein (GCV).

The accuracy rate of CTA in the detection of intracranial circulatory arrest was 100%. CTA examinations confirmed BD diagnoses in all patients who had clinical and EEG BD diagnoses, and no conflict between CTA findings and clinical diagnoses was observed.

## Discussion

BD is defined as the irreversible cessation of all functions of the entire brain, including the cerebrum and brainstem. Based on medical achievements in organ transplant, the diagnosis of BD is of particular importance [1]. It is principally diagnosed using clinical criteria and confirmed by paraclinical means. In most protocols, clinical diagnosis is determined by two independent examiners upon the presence of all the following characteristics: presence of deep unresponsive coma, the absence of all brainstem reflexes (wide open and non-reactive to light pupils, no corneal, cough, and oculocephalic reflexes, no reaction to trigeminal pain stimuli), and the absence of spontaneous respiration after a carbon dioxide (CO2) challenge (‘apnea test’) [1-2]. Some also suggest repeating the clinical evaluation after a 12 - 72-hour period [2]. Moreover, to make the diagnosis of BD, the reversible causes of the comatose state should also be excluded, which include hypothermia (including therapeutic hypothermia), prescription of CNS suppressant drugs, acquired or therapeutic neuromuscular paralysis, high cervical spine injury, and severe acid-base, electrolyte, or endocrine abnormality [1].

Followed by clinical diagnosis of BD, lack of brain function (no cerebral electrical activity) or circulation (‘cerebral circulatory arrest’) should be demonstrated by standard and accepted paraclinical ancillary methods; among them are EEG, TCD, and conventional angiography [3].

Demonstrating cerebral circulatory arrest is a necessity for confirmation of BD. For many years, conventional catheter angiography has been considered the gold-standard method for detection of cerebral circulatory arrest, with blood flow termination at the level of the foramen magnum for posterior circulation and carotid siphon for anterior circulation as the characteristic finding [2, 9]. CTA has emerged as an alternative method [1, 3-4], based on its accessibility in almost all hospitals, high spatiotemporal resolution, lower cost, and operator independence [2, 5-6]. However, an international consensus about the use and parameters of this technique is currently not established [6].

Most series have shown that CTA has demonstrated high sensitivity, specificity, and validity in detecting intracranial circulatory arrest [6]. Different studies have illustrated different CTA criteria for the diagnosis of BD. Some have advocated intracranial non-filling (ICA beyond the level of the anterior clinoid process, VA beyond their dural penetration, ICV, GCV, and the straight sinus) as an indicator of BD. In two studies from the same group, termination of contrast flow at the level of the skull base in the arterial phase and lack of venous blood return in ICVs was observed in BD with > 90% specificity and 97% sensitivity, and the results correlated well with EEG and TCD [5-6]. Some other studies have proposed a seven-vessel score (lack of opacification in both pericallosal arteries (ACA-A3), both cortical segments of the MCA arteries (MCA-M4), both ICVs, and the GCV) [1, 14]. Among these, the most sensitive and earliest sign is the absence of ICVs and MCA-M4 and constitutes the best criterion of BD diagnosis by CTA [3]. Therefore, a four-point scale (lack of opacification in both MCA-M4 and ICVs) has been recommended and used with high sensitivity and specificity [3-4]. In general, a meta-analysis, including eight studies with 337 patients in total, demonstrated a sensitivity estimate of 84 - 85% (no specificity could be calculated and reported) and suggested that, although the available evidence cannot support the use of CTA as a complete replacement for neurological testing, it may be useful as a confirmatory test following a clinical diagnosis of BD [15].

On the other hand, some studies have suggested that CTA is not a validated confirmatory test for cerebral circulatory arrest in brain death, as it may be associated with false-positive results [7] and lower sensitivity compared to EEG [8]. In another study comparing CTA and conventional angiography, inferior results were observed for CTA. However, the authors proposed that divergence of CTA with cerebral angiography was significant mainly concerning proximal ACAs (A2), and confining the interpretation to MCA-M4, PCA-P2, basilar artery, and venous blood return would significantly increase the yield [9]. Others have recommended performing CTA accompanied by CT perfusion, which can be done in the same session and setting, in order to increase the diagnostic yield [10-11]. Moreover, other modalities used in the diagnosis of BD may also contain pitfalls. For instance, EEG cannot be performed or may be difficult to interpret in patients with hypothermia, severe metabolic disorders, drug intoxication, or hemodynamic instability [16], and TCD is operator-dependent and deficient for posterior circulation [17-18].

In the current study, we first confirmed BD in nine patients, mostly victims of traumatic brain injury, with clinical criteria (which were performed twice) and EEG. We then performed a 16-slice CTA. The major observed finding in all patients was a circulatory arrest in both the anterior (beyond the level of skull base and the petrous segment of ICAs) and posterior circulations and also venous return, while external cerebral circulation (STA, for instance) was preserved. The only remnant flow that was observed in a minority of patients was the filling of a short segment of the proximal intradural VA (proximal V4), which does not exclude BD. This finding is similar to intracranial non-filling observed in some other studies [2, 5-6, 19] and simulates the characteristic findings observed in conventional angiography [2, 9]. This finding may yield to 100% specificity for the diagnosis of BD. No sensitivity can be estimated based on our results; nonetheless, regarding the current literature, it may be associated with lower sensitivity compared to other scores. The results correlated completely with a cerebral isoelectric EEG. These results suggest that in a patient with clinical diagnosis of BD, observation of intracranial circulatory arrest or non-filling can confirm the diagnosis of BD. A recent study performed in 18 patients showed a 75% sensitivity, a specificity and positive predictive value of 100%, and a 33% negative predictive value for CTA [20]. The 100% specificity observed in ours and the other study suggests that when the previously described positive findings are observed on CTA, the diagnosis of BD can reliably be confirmed without the need for further ancillary studies. However, based on the relatively lower sensitivity and negative predictive value, a CTA may miss some BD cases (false-negative) and there would be a need for confirmation by other complimenting modalities (like cerebral angiography) to confirm the diagnosis in cases with 'negative' CTAs or those with other equivocal CTA findings.

Based on these, the results of our study were in complete accordance with previous studies that proposed CTA as a useful ancillary test and an alternative to conventional angiography in the diagnosis of BD. Our study, nevertheless, suffers from limitations; the first limitation is its low patient volume. Second, our study was designed in a single-centric nature; the two need further studies recruiting more patients and more centers. Finally, it is possible that we performed CTA late in the course of BD; therefore, we observed the lack of flow in all intracranial arteries and their segments. Should we have performed the CTA sooner, other findings would also be observed. Of course, an interval of six hours before performing a CTA is recommended to avoid repetitive examinations [3]. However, the exact timing of a CTA in the diagnosis of BD should also be elucidated in further studies.

## Conclusions

Brain CTA can be considered as a reliable modality in demonstrating cerebral circulatory arrest, and hence, the diagnosis of brain death. Its findings, in particular, intracranial non-filling, are associated with a high specificity and correlate well with the results of an EEG.
